# Significant Upregulation of HERV-K (HML-2) Transcription Levels in Human Lung Cancer and Cancer Cells

**DOI:** 10.3389/fmicb.2022.850444

**Published:** 2022-03-10

**Authors:** Caiqin Yang, Xin Guo, Jianjie Li, Jingwan Han, Lei Jia, Hong-Ling Wen, Chengxi Sun, Xiaolin Wang, Bohan Zhang, Jingyun Li, Yujia Chi, Tongtong An, Yuyan Wang, Ziping Wang, Hanping Li, Lin Li

**Affiliations:** ^1^Department of Virology, State Key Laboratory of Pathogen and Biosecurity, Beijing Institute of Microbiology and Epidemiology, AMMS, Beijing, China; ^2^Department of Nutrition and Food Hygiene, School of Public Health, Cheeloo College of Medicine, Shandong University, Jinan, China; ^3^Key Laboratory of Carcinogenesis and Translational Research (Ministry of Education/Beijing), Department of Thoracic Medical Oncology, Peking University Cancer Hospital & Institute, Beijing, China; ^4^Key Laboratory for the Prevention and Control of Infectious Diseases, Department of Microbiological Laboratory Technology, School of Public Health, Cheeloo College of Medicine, Shandong University, Jinan, China; ^5^Department of Clinical Laboratory, Cheeloo College of Medicine, Shandong University, Jinan, China

**Keywords:** lung cancer, HERV-K, cell culture, RT–qPCR, *in situ* hybridization, biomarker, diagnosis

## Abstract

Lung cancer is the second most common cancer worldwide and the leading cause of cancer death in the world. Therefore, there is an urgent need to develop new and effective biomarkers for diagnosis and treatment. Under this circumstance, human endogenous retroviruses (HERVs) were recently introduced as novel biomarkers for cancer diagnosis. This study focused on the correlation between lung cancer and HERV-K (HML-2) transcription levels. At the cellular level, different types of lung cancer cells and human normal lung epithelial cells were used to analyze the transcription levels of the HERV-K (HML-2) *gag*, *pol*, and *env* genes by RT–qPCR. At the level of lung cancer patients, blood samples with background information from 734 lung cancer patients and 96 healthy persons were collected to analyze the transcription levels of HERV-K (HML-2) *gag*, *pol*, and *env* genes. The results showed that the transcriptional levels of the HERV-K (HML-2) *gag*, *pol*, and *env* genes in lung cancer cells and lung cancer patient blood samples were significantly higher than those in the healthy controls, which was also verified by RNAScope ISH technology. In addition, we also found that there was a correlation between the abnormal transcription levels of HERV-K (HML-2) genes in lung cancer patients and the clinicopathological parameters of lung cancer. We also identified the distribution locations of the *gag*, *pol*, and *env* primer sequences on each chromosome and analyzed the function of these loci. In conclusion, HERV-K (HML-2) genes may be a potential biomarker for the diagnosis of lung cancer.

## Introduction

Lung cancer, a bronchogenic malignancy originating from epithelial tissue (Wadowska et al., 2020), is the second most commonly diagnosed cancer and the leading cause of cancer deaths worldwide (Schwartz and Cote, 2016; Siegel et al., 2020). According to the latest global cancer burden data for 2020 released by the International Agency for Research on Cancer (IARC) of the World Health Organization, in 2020, there were 2.2 million new cases of lung cancer worldwide (1.44 million males and 770,000 females), accounting for 11.4% of new cases of cancer (14.3% of males and 8.4% of females). In the same period, the global number of lung cancer deaths was 1.8 million (1.19 million males and 610,000 females), accounting for 18% of cancer deaths (21.5% of males and 13.7% of females). The morbidity and mortality of males are almost twice those of females (Sung et al., 2021). The number of deaths from lung cancer is expected to increase to 3 million by 2035 (Siegel et al., 2015; Didkowska et al., 2016). Traditionally, lung cancer is divided into non-small cell lung cancer (NSCLC) and small cell lung cancer (SCLC; Howlader et al., 2020). The most common type of NSCLC includes three histological subtypes: adenocarcinoma (~50%), squamous cell carcinoma (~35%), and large cell carcinoma (~15%; Cagle et al., 2013).

The diagnosis of lung cancer is mainly carried out through imaging technology and pathological examination, both of which have limitations in the comprehensive description of lung cancer (Patz et al., 2014; Konopka, 2016), with only 10–15% of new cases being diagnosed at an early clinical stage (Xi et al., 2018). Therefore, in most cases, lung cancer patients are diagnosed with locally advanced or metastatic diseases (Santarpia et al., 2018), and their prognosis is relatively poor, with 5 year survival rates ranging from 4 to 17% depending on stage and regional differences (Hirsch et al., 2017). In addition, some studies have identified biomarkers associated with lung cancer (Seijo et al., 2019) that can be used in disease diagnosis and treatment response, such as carcinoembryonic antigen (CEA; Hammarström, 1999) and cytokeratin fraction 21-1 (CYFRA 21-1; Shirasu et al., 2018). It is mainly used to help and monitor the treatment process. Unfortunately, these two detection methods can only cover a small number of patients with NSCLC. At this stage, we cannot achieve the prevention and early detection of lung cancer through the determination of biomarkers (Cagle et al., 2013). Therefore, in lung cancer, it is necessary to further understand the changes in other genes. Finding high-efficiency lung cancer biomarkers and their clinical application is one of the important means to improve the early diagnosis rate and treatment effectiveness of lung cancer. Some achievements have been made in this field, but the specificity and sensitivity of the verified lung cancer biomarkers still need to be further improved.

Human endogenous retroviruses (HERVs) are novel biomarkers that have recently been introduced for cancer diagnostic purposes (Schmitt et al., 2015; Mareschi et al., 2019; Cao et al., 2020; Curty et al., 2020). HERVs are remnants of retroviruses that infected humans millions of years ago and are integrated into the cellular genome, comprising approximately 8% of the human genome, and are inherited in a Mendelian fashion, with a gene structure consisting of the *gag*, *pro*, *pol*, and *env* genes (Lander et al., 2001; Balestrieri et al., 2015; Johanning et al., 2017; Gruchot et al., 2019). According to their genetic origin, HERVs can be divided into three main groups: ① ERV-Class I: HERV-W, HERV-R, HERV-E, HERV-H, ERV-9, and HERV-I; ② ERV-Class II: HERV-K; and ③ ERV-Class III: HERV-S and HERV-L (Kim et al., 2008; Escalera-Zamudio and Greenwood, 2016; Vargiu et al., 2016). Through the process of recombination, deletion, and inactivation mutations (Hughes and Coffin, 2001; Lander et al., 2001; Grandi et al., 2017; Grandi, 2018), HERVs are inserted into the human genome in the form of gene fragments, weakening their ability to form infectious virus particles (Blomberg et al., 2009; Dewannieux and Heidmann, 2013), but HERV elements can be transcriptionally active, and many sequences retain open reading frames capable of producing viral proteins (de Parseval et al., 2003; Büscher et al., 2006). HERV-K is the HERV with the most complete viral genetic structure and is capable of producing biologically active retroviral-like particles (Medstrand and Mager, 1998; Buzdin et al., 2003). In recent years, it has been found that abnormal expression of HERV-K proteins and reverse transcription elements may lead to the development of pathological states (Zhou et al., 2016; Lemaitre et al., 2017). HERV-K RNA expression and protein products are widely present in a variety of malignancies (Büscher et al., 2006; Ehlhardt et al., 2006; Fuchs et al., 2011). A study comparing the mRNA expression of HERVs in tumors and adjacent normal tissues showed that HERV-R and H were expressed at high levels in lung tumor tissue compared to normal adjacent tissue (Ahn and Kim, 2009). Another study showed that the blood levels of HERV-R, HERV-H, HERV-K, and HERV-P *env* mRNA in lung cancer patients were significantly higher than those in healthy controls, and the level of HERV *env* mRNA in patients with adenocarcinoma was generally higher than that in patients with squamous cell carcinoma and SCLC (Zare et al., 2018).

The above studies have proven that the abnormal expression of HERV-K *env* is closely related to the occurrence of lung cancer, but there is no related literature on the analysis of HERV-K *gag* and *pol* transcription levels in PBMCs of lung cancer patients. In this case, it is necessary to analyze the transcriptional levels of HERV-K genes in lung cancer patients. Therefore, in this study, lung cancer cell lines and control cells, lung cancer patients, and healthy individuals were selected as subjects to analyze the transcriptional levels of HERV-K (HML-2) *gag*, *pol*, and *env* genes based on RT–qPCR technology and to analyze their correlation with the occurrence and development of lung cancer. Our results show that the occurrence of lung cancer is associated with HERV-K (HML-2) *gag*, *pol*, and *env*, indicating that HERV-K (HML-2) genes may be a potential biomarker for the diagnosis of lung cancer.

## Materials and Methods

### Patients and Samples

Blood samples were collected in tubes containing dipotassium EDTA anticoagulant from 734 lung cancer patients, including 356 adenocarcinomas, 159 squamous cell carcinoma, 17 large cell carcinoma, and 157 small cell lung cancer; the rest were unknown. According to the disease stage, there were 35 cases of I–II and 213 cases of III-IV, and according to the treatment protocol, there were 110 cases without treatment, 414 cases with CTx, and 185 cases with CTx + RTx. Additionally, 96 age- and sex-matched individuals without a history of tumors, autoimmune diseases, or neurological diseases were selected as healthy controls. Written informed consent was obtained from all lung cancer patients and healthy individuals, and the data were analyzed anonymously. All samples were transported to our laboratory through a cold chain and stored at −80°C. The demographic and basic information of the participants is presented in Table 1.

**Table 1:** Study’s characteristics between the patients and healthy controls.

Characteristics	Categories	Cases		Controls	
		*n* = 734	Mean ± SD	*n* = 96	Mean ± SD
Age (Year)^1^	20–40	25 (3.41%)	61.20 ± 9.82	32 (33.33%)	48.24 ± 16.65
	41–60	283 (38.56%)		39 (40.63%)	
	61–80	411 (56.00%)		23 (23.96%)	
	81–100	8 (1.09%)		2 (2.08%)	
Gender^2^	Male	499 (67.99%)	–	65 (67.71%)	–
	Female	235 (32.02%)	–	31 (32.30%)	–
Type of cancer^2^	SCLC	157 (21.39%)	–	–	–
	Adenocarcinoma	356 (48.50%)	–	–	–
	Squamous cell carcinoma	159 (21.66%)	–	–	–
	Large cell carcinoma	17 (2.32%)	–	–	–
Disease stage^2^	I–II	35 (4.77%)	–	–	–
	III–IV	213 (29.10%)	–	–	–
Chemotherapy protocol^2^	Treatment naive	110 (14.99%)			
	CTx	414 (56.40%)			
	CTx + RTx	185 (25.20%)			

1 Mean ± SD for quantitative variables.

2 Number (Relative Frequency %) for the categorical variable.

### Cell Lines and Cell Culture

Human lung bronchial epithelial BEAS-2B cells, human small cell lung cancer NCI-H446 cells, human adenocarcinoma Calu-3 cells, human squamous cell carcinoma SK-MES-1 cells, and human large cell carcinoma NCI-H460 cells were obtained from Shanghai Chinese Academy of Sciences Cell Bank (Shanghai, China) and Procell (Wuhan, China). The cell lines BEAS-2B, NCI-H446, Calu-3, SK-MES-1, and NCI-H460 were cultured in RPMI 1640, DMEM/F12, or MEM (Gibco, United States) with 10% fetal calf serum (Gibco, United States), 100 U/ml penicillin, and 100 μg/ml streptomycin at 37°C with 5% CO_2_ and saturated humidity.

### RNA Preparation and cDNA Synthesis

Total RNA was extracted using the MiniBEST Plant RNA Extraction Kit (TaKaRa, Cat No. 9769) according to the manufacturer’s protocol. To remove genomic DNA, all RNA samples were treated with 1 μl of gDNA eraser per 1 μg of RNA (TaKaRa, Cat No. RR047A). Then, real-time quantitative PCR was used to detect DNA contamination in all RNA samples, which showed that the CT value of β-actin was >40 in all samples. To further assess whether these gDNA-erased samples had any residual DNase activity or possible inhibition of PCR, we added 10^3^ and 10^2^ DNA standards to the treated samples or pure water controls and compared their CT values. We also performed double gDNA eraser treatments on the low-copy RNA standards, and we found no evidence of RNA copy loss, as there was no change in CT values for successive treatments (Hurst et al., 2016; Karamitros et al., 2016). Then, the mRNA was reverse transcribed into cDNA using a mixed primer pair that contained Oligo dT primer according to the manufacturer’s protocol (TaKaRa, Cat No. RR047A): 37°C for 15 min and 85°C for 5 s, followed by storage at 4°C.

### Real-Time Quantitative Polymerase Chain Reaction

Real-time quantitative PCR was performed with a Roche LightCycler 480 II System, 96-well format, using TB Green® Premix Ex Taq™ (TaKaRa, Cat No. RR420A) in a 20 μl reaction with 2 μl cDNA and final concentrations of 0.25 μM forward primer and 0.25 μM reverse primer. All reactions were run using the following protocol: cycling conditions were a 10 min denaturation step at 95°C, followed by 40 cycles of 10 s at 95°C, 5 s at 60°C, and 10 s at 72°C. At the end of the reaction, the amplification curve and melting curve were analyzed. The presence of a single peak in the melting curve analysis was applied to validate the specificity of the PCR amplification. For each amplicon, cDNA samples were measured in triplicate. Primer sequences for amplification of HERV-K (HML-2) *gag*, *pol*, and *env* and β-actin were obtained from a previous study (Li et al., 2021). These sequences were checked by the Primer Blast, Oligocalc, and Gene Runner programs. Primer information is shown in Table 2.

**Table 2:** Primers of human endogenous retroviruses (HERVs) genes for real-time PCR analysis.

Target gene	Primer sequence (5'–3')	Direction
β-*actin* F	CCACGAAACTACGTTCAACTCC	Forward
β-*actin* R	GTGATCTCCTTCTGCATCCTGT	Reverse
HERV-K *gag* F	AGCAGGTCAGGTGCCTGTAACATT	Forward
HERV-K *gag* R	TGGTGCCGTAGGATTAAGTCTCCT	Reverse
HERV-K *pol* F	TCACATGGAAACAGGCAAAA	Forward
HERV-K *pol* R	AGGTACATGCGTGACATCCA	Reverse
HERV-K *env* F	CTGAGGCAATTGCAGGAGTT	Forward
HERV-K *env* R	GCTGTCTCTTCGGAGCTGTT	Reverse

### Quantification of HERV-K (HML-2) Transcription in Cells by RNAscope® ISH Technology

RNAscope® (RNAscope® Multiplex Fluorescent Reagent Kit v2, Advanced Cell Diagnostics, United States, Cat No. 323100) was used according to the manufacturer’s protocol and was adapted for the dual detection of mRNA. The probe sets HERV-K (HML-2) *env*-C1, HERV-K (HML-2) *pol*-C2, or HERV-K (HML-2) *gag*-C3 (Advanced Cell Diagnostics, United States) consisted of 20 dual probes targeting different segments within the HERV-K (HML-2) *env*, HERV-K (HML-2) *pol*, or HERV-K (HML-2) *gag*. The standard sample was pretreated according to the manufacturer’s instructions and the previously reported literature (Wang et al., 2012; Li et al., 2021). After the cells were treated, they were inoculated on culture slides. The slide was dipped into 10% neutral buffer formalin (NBF) and fixed at room temperature (RT) for 30 min. Pretreatment for storage was performed by dehydration using 50, 70, and 100% ethanol for 5 min at room temperature and then rehydration at room temperature with 100, 50, and 70% ethanol. The slide was removed from 1× PBS, and a hydrophobic barrier was created by using an Immedge™ Hydrophobic Barrier Pen (Vector Laboratory, United States). The slide was then incubated with hydrogen peroxide and protease (RNAscope® H_2_O_2_ and Protease Reagents, Cat No. 322281) at 40°C for 10 and 30 min, respectively. The probe was mixed according to the dose requirement. Then, the mixed target probes were incubated with a HyBez™ oven (Advanced Cell Diagnostics, 110 V, Cat No. 310010) at 40°C for 2 h, and the corresponding probes were added dropwise to the negative (RNAscope® 3-plex Negative Control Probe, Cat No. 320871) and positive (RNAscope® 3-plex Positive Control Probe-Hs, Cat No. 320861) controls. The signal was amplified by incubating one drop each of AMP-1, AMP-2, and AMP-3 at 40°C for 30, 30, and 15 min, respectively, using a HyBez™ oven. Each target nucleic acid was fluorescence-stained with Opal™ 520 (PerkinElmer, Cat No. FP1487001KT), Opal™ 570 (PerkinElmer, Cat No. FP1488001KT), and Opal™ 690 (PerkinElmer, Cat No. FP1497001KT) for 30 min at 40°C and washed twice with wash buffer. Prolong Gold Antifade Mountant (Prolong™, Invitrogen, Cat No. P10144) was used to prevent fluorescence quenching. Then, the coverslips were covered, and a picture was taken as soon as possible. The excitation/emission and pass wavelengths used to detect DAPI, Opal™ 520, Opal™ 570, and Opal™ 690 were set to 340–370/410–470, 460–480/490–530, 510–550/570–590, and 630–650/640–670 nm, respectively. Superresolution images were captured using a confocal microscope (NIKON TI2-E; CRESTOPTICS X-LIGHT V3).

### Bioinformatic Analyses

In this study, bioinformatic analyses were performed to predict the chromosome distribution loci and related functions of the HERV-K (HML-2) *gag*, *pol*, and *env* genes. Based on HERV-K (HML-2) *gag*, *pol*, and *env* gene primer sequences, the UCSC genome browser in genome assembly HG38^1^ Blat is used to quickly search the chromosomal coordinates of short nucleotide or protein sequences encoded within the genome. The functional role of these chromosomal loci was then analyzed using the GREAT tool^2^ and WebGestalt.^3^

### Statistical Analysis

Statistical analysis was performed using GraphPad Prism 8 (GraphPad, CA, United States) and SPSS version 25.0 (SPSS Inc., IL, United States). The Shapiro–Wilk test was applied to test for a normal distribution. Normally distributed numerical variables were expressed as the mean ± standard deviation (SD) taken from at least three independent experiments using an unpaired Student’s *t*-test. *p* values <0.05 were considered significant, and ^*^p < 0.05, ^**^p < 0.01, ^***^p < 0.001, and ^****^p < 0.0001.

## Results

### Expression of HERV-K (HML-2) *gag*, *pol*, and *env* in the Lung Cancer Cell Lines NCI-H446, Calu-3, SK-MES-1, and NCI-H460 by RT–qPCR

**Figure 1:**
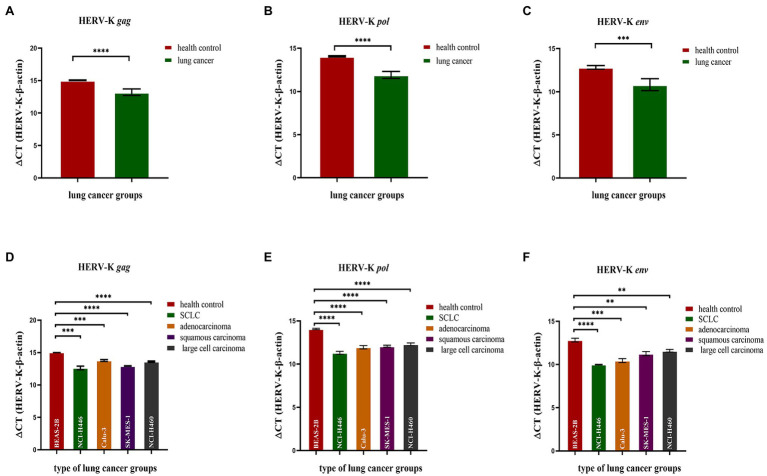
Analysis by real-time quantitative RT–PCR of HERV-K (HML-2) *gag*, *pol*, and *env* gene expression in the different human lung cancer cell lines. The expression of the HERV-K (HML-2) *gag* **(A,D)**; HERV-K (HML-2) *pol* **(B,E)**; and HERV-K (HML-2) *env* **(C,F)** genes in the lung cancer cell lines NCI-H446, Calu-3, SK-MES-1, NCI-H460, and human bronchial epithelial BEAS-2B cells was analyzed. Real-time quantitative RT–PCR was performed with cell total RNA from the different human lung cancer cell lines, including the healthy control groups, lung cancer groups and lung cancer groups. Real-time quantitative RT-PCRs were coupled to melting-curve analysis to confirm the amplification specificity. Nontemplate controls were included for each primer pair to check for any significant levels of contaminants. The relative expression levels of the HERV-K (HML-2) *gag*, *pol*, and *env* genes were normalized to the expression level of the β-actin gene. Experiments were repeated three times to ascertain the reproducibility of the results. The *Y*-axis is ΔCT = CT_HERV−K_−CT_β−actin_. *p < 0.05, **p < 0.01, ***p < 0.001, ****p < 0.0001.

We analyzed the transcriptional levels of HERV-K (HML-2) *gag*, *pol*, and *env* mRNA in the lung cancer cell lines NCI-H446, Calu-3, SK-MES-1, NCI-H460, and BEAS-2B by RT–qPCR. The transcriptional levels of the HERV-K (HML-2) *gag*, *pol*, and *env* genes in lung cancer were significantly higher than those in the healthy controls (*p* < 0.0001, Figures 1A–C). Analysis of the expression of HERV-K (HML-2) *gag, pol*, and *env* in lung cancers showed that the transcriptional levels of *gag*, *pol*, and *env* mRNA were significantly higher in SCLC, adenocarcinoma, squamous cell carcinoma, and large cell carcinoma than in healthy controls (Figures 1D–F).

### Expression of HERV-K (HML-2) *gag*, *pol*, and *env* in Lung Cancer Cell Lines Was Assessed by the RNAscope® ISH Technique

**Figure 2:**
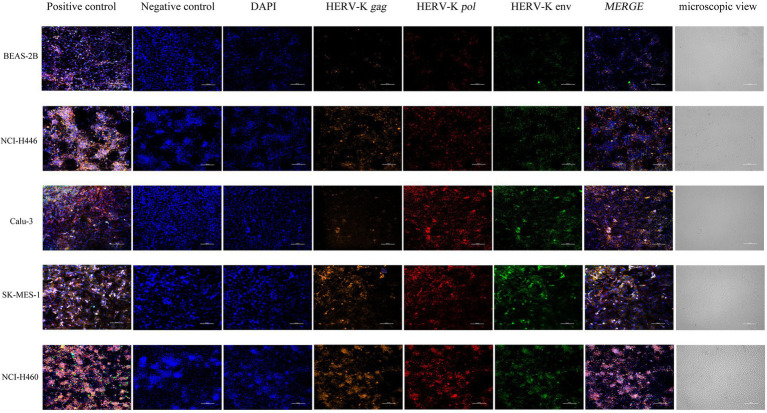
Analysis by RNAscope® ISH technology of HERV-K (HML-2) *gag*, *pol*, and *env* gene expression in the different human lung cancer cell lines. DAPI was used to stain and label the nuclei, Opal™ 520 fluorescent dye with a special probe was used to stain and label HERV-K (HML-2) *env* region mRNA (green), Opal™ 570 fluorescent dye with a special probe was used to stain and label HERV-K (HML-2) *pol* region mRNA (red), and Opal™ 690 fluorescent dye with a special probe was used to stain and label HERV-K (HML-2) *gag* region mRNA (orange). The positive and negative controls used standard control probes provided in the kit (ACD). Original magnification: 20×; scale bars: 100 μM.

We used NCI-H446, Calu-3, SK-MES-1, NCI-H460, and BEAS-2B cells to show the *in situ* HERV-K (HML-2) *gag*, *pol*, and *env* RNA expression sites and relative abundance using RNAScope® ISH technology. Sections were made when the cell density was 80%. At the same time, positive control and negative control sections were prepared. Compared with the healthy controls, the transcriptional levels of HERV-K (HML-2) *gag*, *pol*, and *env* in the lung cancers were significantly increased (Figure 2). The experimental results of RNAScope® are consistent with the results of RT–qPCR at the cellular level.

### Expression of HERV-K (HML-2) *gag*, *pol*, and *env* in the Blood of Lung Cancer Patients by RT–qPCR

**Figure 3:**
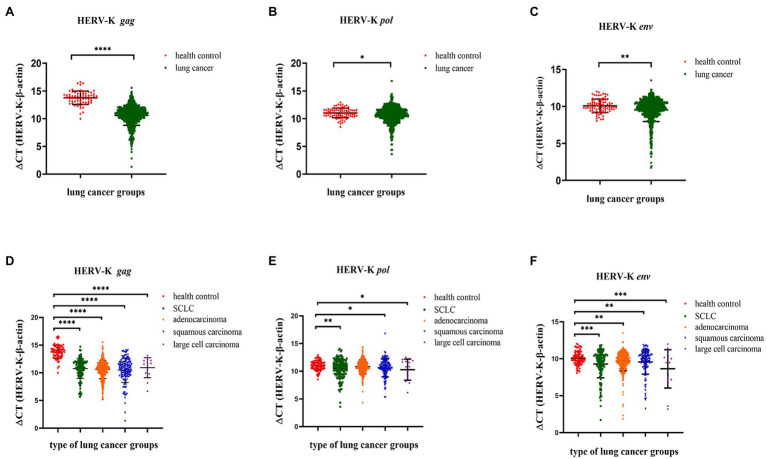
Analysis by real-time quantitative RT–PCR of HERV-K (HML-2) *gag*, *pol*, and *env* gene expression in the different lung cancer patients. The expression of the HERV-K (HML-2) *gag* **(A,D)**; HERV-K (HML-2) *pol* **(B,E)**; and HERV-K (HML-2) *env* **(C,F)** genes in the different lung cancer patients was analyzed. Real-time quantitative RT-PCRs were performed with blood total RNA of the different lung cancer patients, including healthy control groups, lung cancer groups and lung cancer groups. Real-time quantitative RT-PCRs were coupled to melting-curve analysis to confirm the amplification specificity. Nontemplate controls were included for each primer pair to check for any significant levels of contaminants. The relative expression levels of the HERV-K (HML-2) *gag*, *pol*, and *env* genes were normalized to the expression level of the β-actin gene. Experiments were repeated three times to ascertain the reproducibility of the results. The *Y*-axis is ΔCT = CT_HERV−K_−CT_β−actin_. *p < 0.05, **p < 0.01, ***p < 0.001, ****p < 0.0001.

We collected background information and blood samples from 734 lung cancer patients and 96 healthy controls and then analyzed the transcriptional levels of the HERV-K (HML-2) *gag*, *pol*, and *env* genes in different lung cancer patients by RT–qPCR. The transcriptional levels of HERV-K (HML-2) *gag*, *pol*, and *env* in lung cancers were significantly upregulated compared with those in healthy controls (*p* < 0.0001, *p* < 0.05, and *p* < 0.01, respectively, Figures 3A–C). Analysis of HERV-K (HML-2) *gag*, *pol*, and *env* gene transcription in lung cancers showed that the transcriptional levels of *gag*, *pol*, and *env* mRNA were significantly higher in SCLC, adenocarcinoma, squamous cell carcinoma, and large cell carcinoma than in healthy controls (Figures 3D–F).

### Correlation Between HERV-K (HML-2) *gag*, *pol*, and *env* and the Pathologic Features of Lung Cancer

**Figure 4:**
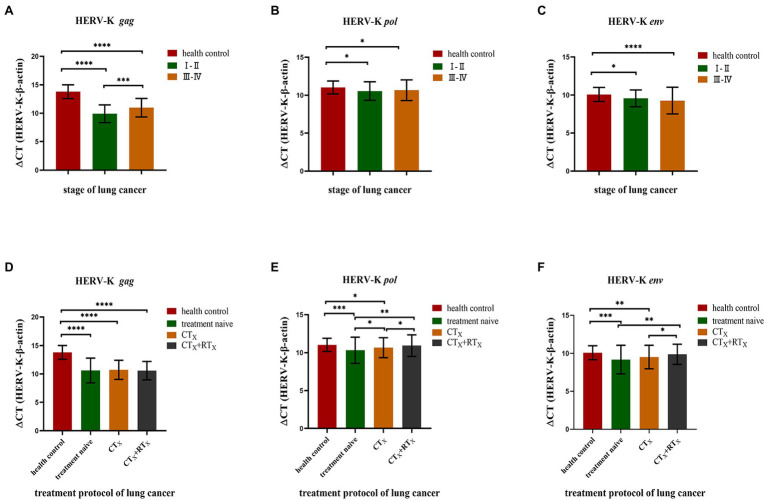
Analysis of HERV-K (HML-2) *gag*, *pol*, and *env* gene expression according to various stages of lung cancer and treatment protocols. **(A)** HERV-K (HML-2) *gag*, *pol*, and *env* gene transcript levels in the blood of patients with stage I–II and III–IV lung cancer. **(B)** Effect of chemotherapy and cotreatment with radiotherapy on the reduction of HERV-K (HML-2) *gag*, *pol*, and *env* genes in the blood of lung cancer patients. The expression of the HERV-K (HML-2) *gag*, *pol*, and *env* genes was compared by chemotherapy method, including treatment naive, chemotherapy included (CTx), and chemotherapy accompanying radiotherapy (CTx + RTx) groups. The *Y*-axis is ΔCT = CT_HERV−K_−CT_β−actin_. *p < 0.05, **p < 0.01, ***p < 0.001, ****p < 0.0001.

We found a significant correlation between HERV-K (HML-2) and some pathologic data, such as stage and treatment protocol. Our results showed that the transcriptional levels of the HERV-K (HML-2) *gag*, *pol*, and *env* genes in different stages of lung cancer (stage I–II and stage III–IV) were higher than those in healthy controls (Figure 4A). There was also a significant difference in the transcriptional levels of the HERV-K (HML-2) *gag* gene between I-II and III-IV (*p* < 0.01). However, there was no significant difference in the transcriptional levels of HERV-K (HML-2) *pol*, *env* between I-II and III-IV. We compared treatment naive, chemotherapy included (CTx), and chemotherapy accompanying radiotherapy (CTx + RTx) and found that the transcriptional levels of HERV-K (HML-2) decreased in the chemotherapy groups, while the transcriptional levels of HERV-K (HML-2) in the chemotherapy with radiotherapy groups approached those of healthy controls (Figure 4B). The results suggest that the combination therapy is conducive to reducing the expression of HERV-K (HML-2) genes.

### GO Annotation and KEGG Pathway Enrichment Analyses

**Figure 5:**
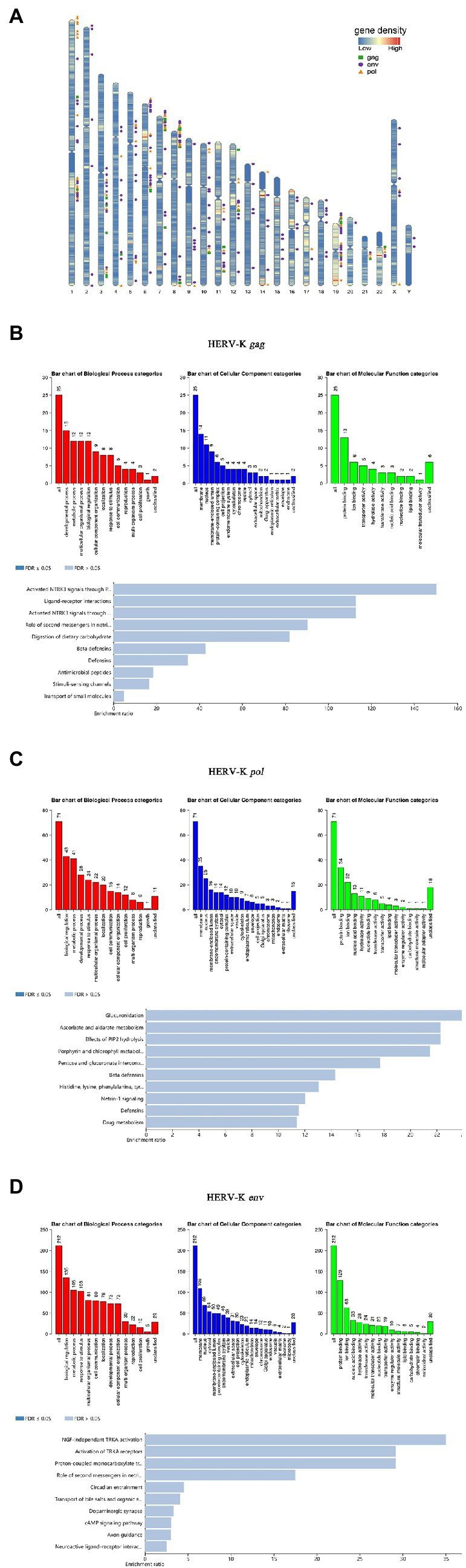
Chromosomal distribution of HERV-K (HML-2) loci and GO analyses. **(A)** HERV-K (HML-2) gene loci have been visualized on the human karyotype; the green square is HERV-K (HML-2) *gag*, the orange triangle is HERV-K (HML-2) *pol*, and the purple circle is HERV-K (HML-2) *env*. **(B**-**D)** GO annotation and KEGG pathway enrichment analyses. All host genes with transcript expression profiles correlated with HERV-K (HML-2) *gag*, *pol*, and *env* abundance were analyzed using the GREAT and WebGestalt tools for enriched GO terms. Each biological process, cellular component and molecular function category is represented by a red, blue, and green bar, respectively. The height of the bar represents the number of IDs in the user list and in the category. Using WebGestalt bioinformatics resources, the predicted target genes were classified according to KEGG functional annotations to determine the regulation pathway of HERV-K (HML-2) *gag*, *pol*, and *env* transcripts.

In this study, bioinformatic analyses were performed to predict the chromosome distribution loci. Based on HERV-K (HML-2) *gag*, *pol*, and *env* gene primer sequences, the UCSC genome browser in genome assembly HG38 Blat was used to quickly search the chromosomal coordinates (Figure 5A). To obtain deeper insight into the biological roles of these loci, GREAT and WebGestalt were employed to conduct Gene Ontology (GO) annotation and Kyoto Encyclopedia of Genes and Genomes (KEGG) pathway enrichment analyses. The top 10 enriched GO terms and KEGG pathways are shown in this study. Regarding HERV-K *gag*, GO biological process (BP) analysis revealed that these loci were markedly enriched in the developmental process. For GO cellular component (CC) analysis, the top significantly enriched term was membrane. The top significantly enriched molecular function (MF) term was protein binding. In addition, the top markedly enriched pathway for these *gag* loci was the activated neurotrophic receptor tyrosine kinase 3 (NTRK3) signal (Figure 5B). It is worth noting that the GO analysis of *pol* and *env* was similar. GO BP analysis revealed that the *pol* and *env* loci were markedly enriched in biological regulation. For GO CC analysis, the top significantly enriched term was membrane. The top significantly enriched MF term was protein binding. In addition, the top markedly enriched pathway for these *pol* loci was glucuronidation. The top markedly enriched pathway for these *env* loci was nerve growth factor (NGF)-independent tyrosine kinase (TRKA) (Figures 5C,D). The genes used for GO and KEGG analysis are listed in Supplementary Table S1 and the associations between each HERV-K (HML-2) and the genes it putatively regulates are shown in Supplementary Figure S1.

## Discussion

Lung cancer has the characteristics of concealed pathogenesis, rapid progression, poor prognosis, and high mortality, which lead to tumor invasiveness, frequent intrapulmonary spread and extrapulmonary metastasis, and frequent postoperative recurrence. Due to the lack of effective early clinical diagnosis, only a small number of lung cancer patients can undergo potential radical resection, which can improve the 5 year survival rate. Therefore, to improve the diagnostic accuracy and therapeutic effect of lung cancer, it is essential to explore new and effective tumor biomarkers and their functional mechanism. In this study, we found that the transcription levels of HERV-K (HML-2) *gag*, *pol*, and *env* genes in lung cancer cells and lung cancer patients were significantly higher than those in healthy controls, showing that the occurrence of lung cancer is associated with the HERV-K (HML-2) *gag*, *pol*, and *env* genes, suggesting that HERV-K (HML-2) genes may be a potential biomarker for the diagnosis of lung cancer.

Human endogenous retrovirus genetic elements make up approximately 8% of the human genome and are distributed comprehensively in the human chromosome (Kurth and Bannert, 2010). Originally, they were considered “junk DNA.” Growing evidence has proven that they might be involved in certain physiological and pathological processes (Jeong et al., 2010; Romanish et al., 2010). For example, the Env protein of HERV-W has been confirmed to induce inflammation in patients with multiple sclerosis through the toll-like receptor 4 (TLR4) activation pathway (Perron et al., 2013). In addition, recent studies have shown that HERVs transcriptional activity is significantly associated with cancers (Matteucci et al., 2018). Furthermore, there are HERVs (mainly HERV-K) virus-like particles, transcripts, and proteins in different types of human cancers, as well as their specific antibodies in the sera of patients. Dube et al. (2014) found HERV-K viral particles in teratocarcinoma cell lines and patient plasma, and HERV-K can initiate reverse transcription activity of reverse transcriptase to convert RNA to DNA and infest host cells. Wang-Johanning et al. (2014) showed that high expression of HERV-K (HLM-2) was detected in the serum of patients with early breast cancer, and the level of mRNA was positively correlated with the aggravation of clinical manifestations of breast cancer. Fava et al. (2016) found that high expression of HERVs was also found in peripheral monocytes of patients with T-cell lymphoma, suggesting that it was abnormal in the early stage of the disease. Rycaj et al. (2015) studies showed that the level of HERV-K antibody increased significantly in the blood of patients with ovarian cancer and stimulated the immune response, viral T-cell proliferation, elevated levels of interferon-γ, and increased the activity of antigen-specific viral L cells. HERV activity is strictly regulated in the life cycle of each individual (Balestrieri et al., 2015) and is related to the state of chromatin (van de Lagemaat et al., 2006). Many mechanisms have evolved to silence the activity of HERVs. However, a very small number of HERVs are unstable and still active. Their reproduction and random insertion of cell DNA will lead to genetic changes, resulting in abnormal expression in a variety of diseases (Wildschutte et al., 2016).

Aberrant activation of HERV genes is a promising field of diagnosis and treatment in cancer research. HERV genes or gene products show pathogenic potential in a variety of human diseases, such as autoimmune diseases, neurological diseases, infectious diseases, and cancer (Suntsova et al., 2015). Compared with healthy controls, the increased transcriptional expression of HERV-K in malignant diseases such as colorectal cancer, breast cancer, liver cancer, melanoma, and prostate cancer has been studied. Pérot et al. (2015) specifically detected several gene loci of HERV-K and found that the transcriptional levels of HERV-K can stably reflect the progression of colorectal cancer and help to improve the sensitivity and specificity of clinical detection of colorectal cancer. The detection frequency of HERV-specific transcripts in the brain tissue of ALS patients was higher than that of non-ALS control patients (刘剑凯, 2008). The levels of HERV-K mRNA expression and the titer of anti-HERV-K antibody in the serum of patients with ductal carcinoma *in situ* or stage I breast cancer were significantly higher than those of normal controls, and those with metastasis were higher than those without metastasis, suggesting that it can be used as a serum biomarker for early breast cancer diagnosis (Wang-Johanning et al., 2014). With a large differential expression profile in hepatoblastoma and very low mRNA levels in liver control samples, HERV-K mRNA expression may be a useful biomarker in hepatoblastoma (Grabski et al., 2021). The mRNA expression level of the HERV-K family *gag* in peripheral blood mononuclear cells of patients with prostate cancer was significantly higher than that of healthy people, especially in elderly individuals and smokers (Wallace et al., 2014). As mentioned above, although HERV-K has been evaluated and introduced as a potential biomarker for breast cancer and melanoma, there are no systematic studies to evaluate the ability of the HERV-K gene to diagnose lung cancer. To clearly evaluate the correlation between HERV-K and development of lung cancer, this study focused on the correlation between lung cancer and HERV-K (HML-2) gene transcriptional levels. At the cellular level, SCLC, adenocarcinoma, squamous cell carcinoma, large cell lung cancer, and human normal lung epithelial cells were used to analyze the transcriptional levels of the HERV-K (HML-2) *gag*, *pol*, and *env* genes based on RT–qPCR. The transcriptional levels of the HERV-K (HML-2) *gag*, *pol*, and *env* genes in lung cancer cells were significantly higher than those in normal lung cells. This result was also verified by RNAscope® ISH technology. At the level of lung cancer patients, background information and blood samples were collected from 734 lung cancer patients (157 cases of SCLC, 356 cases of adenocarcinoma, 159 cases of squamous cell carcinoma, and 17 cases of large cell lung cancer) and 96 healthy controls. The transcriptional levels of the HERV-K (HML-2) *gag*, *pol*, and *env* genes in different lung cancer patients were analyzed. The transcriptional levels of the HERV-K (HML-2) *gag*, *pol*, and *env* genes in lung cancer patients were significantly higher than those in healthy controls. The reason why the experimental results at the cellular level and the patient population level are not very consistent may be due to individual differences and lymphocyte enrichment. In addition, interestingly, we found that there was a correlation between the abnormal expression levels of HERV-K (HML-2) genes in blood of lung cancer patients and the clinicopathological parameters of lung cancer, such as the disease stage and treatment protocol of lung cancer. The results showed that the transcriptional levels of the HERV-K (HML-2) *gag*, *pol*, and *env* genes in lung cancer patients with different stages (stage I–II and stage III–IV) were higher than those in healthy controls. Compared with untreated lung cancer patients, it was found that the transcriptional levels of HERV-K (HML-2) genes decreased in lung cancer patients treated with chemotherapy and radiotherapy, while the transcriptional levels of HERV-K (HML-2) in chemotherapy with radiotherapy group tended to be close to that of healthy controls, suggesting that combination therapy is more beneficial to reduce the expression of HERV-K (HML-2) genes.

In this study, we revealed HERV-K (HML-2) loci transcribed in lung cancer. The identified HERV-K (HML-2) loci are of immediate relevance if the HERV-K (HML-2) transcript is considered to play a role in lung cancer. Therefore, in this study, bioinformatics methods were used to predict the chromosome distribution loci and related functions of the HERV-K (HML-2) *gag*, *pol*, and *env* genes. In this study, the results of bioinformatics analysis showed that the number of chromosome loci where the HERV-K (HML-2) *gag*, *pol*, and *env* genes were distributed was 29, 71, and 158, respectively, suggesting that HERV-K (HML-2) genes exist widely in the human chromosome genome. In addition, the results of bioinformatics analysis showed that the top markedly enriched pathway for these HERV-K (HML-2) *gag* loci was the activation of the NTRK3 signal, and the NTRK fusion gene was one of the tumor-driving genes. NTRK gene fusion led to the fusion of NTRK gene family members (NTRK1, NTRK2, and NTRK3) with another unrelated gene. The NTRK fusion protein will be continuously active, triggering a permanent signal cascade that drives the spread and growth of NTRK fusion tumors, including lung cancer (Harada et al., 2021; Konig et al., 2021). Studies have shown that high HERV-K (HML-2) transcription alters cortical layer formation in forebrain organoids, and transcriptional activation leads to hyperactivation of NTRK3 expression and other neurodegeneration-related genes (Padmanabhan Nair et al., 2021). The top markedly enriched pathway for these HERV-K (HML-2) *pol* loci was glucuronidation, and recent studies have shown that glucuronidation increases in malignant tumors (Landmann et al., 2014; Zahreddine et al., 2014). The top markedly enriched pathway for these HERV-K (HML-2) *env* loci was NGF-independent TRKA activation. Tyrosine kinase (TRKA) is the functional receptor of nerve growth factor (NGF), which has been shown to be associated with tumorigenesis, proliferation, angiogenesis, and metastasis. NGF and its receptors are expressed in lung cancer, and this autocrine stimulus plays an important role in the development of malignant tumors and is associated with low tumor survival rates (Prakash et al., 2010). Kim and other studies have shown that TRKA inhibitors have a significant inhibitory effect on the proliferation of human lung cancer cells but have a low inhibitory effect on normal MRC5 cells (Yan et al., 2021). In summary, it is very important to identify specific HERV-K sites related to specific diseases, especially HERV-K polymorphic sites, which may affect the expression profile of the virus in different individuals and the regulation of host genes. HERV-K (HML-2) is closely related to the occurrence and development of lung cancer, so it is necessary to study the loci of HERV-K (HML-2) and its functional mechanism in the progression of lung cancer.

We confirmed that the transcriptional levels of HERV-K (HML-2) were significantly upregulated in lung cancer patients compared with healthy controls and provided insights into the relationship with the clinical characteristics of lung cancer patients. However, our study still has many limitations. First, we only analyzed the transcriptional levels of HERV-K (HML-2) genes in lung cancer patients, but unfortunately, we do not know which loci play a major role. From our research, we can prove that the occurrence of lung cancer is closely related to HERV-K (HML-2) *gag*, *pol*, and *env*, so we consider that HERV-K (HML-2) is a potential biomarker, rather than just identifying it as such. Second, the expression changes of non-HERV genes have not been studied, and it is not possible to link the data to the expression changes that occur in these cancers, which is also a limitation of our study. High-throughput sequencing technology is widely used to find candidate genes for diseases, which can better and more deeply study the interaction between lung cancer and HERV-K (HML-2). Next, we plan to identify specific HERV-K (HML-2) loci with altered transcript levels in lung cancer patients based on high-throughput sequencing in future studies. We will then conduct systematic functional validation experiments based on the specific loci to clarify the important role of the HERV-K (HML-2) gene in the development of lung cancer, and we will try to collect lung cancer tissue samples and control samples for experiments to validate the results against each other.

The biological function and disease association of HERVs are largely elusive. Obviously, more research is needed to understand the role of HERV-K (HML-2) in human lung cancer and to clarify its role in the pathogenesis of the disease, which may provide new ideas for the further discovery of tumor-associated antigens and the development of new treatments for lung cancer.

## Data Availability Statement

The original contributions presented in the study are included in the article/Supplementary Material, further inquiries can be directed to the corresponding authors.

## Ethics Statement

The studies involving human participants were reviewed and approved by Beijing Institute of Microbiology and Epidemiology. The patients/participants provided their written informed consent to participate in this study.

## Author Contributions

CY, LL, and HL contributed to conception and design of the study. JL, XG, and ZW contributed to sample collection. CY contributed to organizing the database, performing the statistical analysis, and writing the first draft of the manuscript. LJ, JH, and XW contributed to the bibliographic survey. BZ and JH were involved in the critical revision of the article. All authors contributed to the article and approved the submitted version.

## Funding

This study was supported by the National Key Research and Development Program of China (2020YFA0907000), the NSFC (81773493, 31800149, and 31900157), and the State Key Laboratory of Pathogen and Biosecurity (AMMS).

## Conflict of Interest

The authors declare that the research was conducted in the absence of any commercial or financial relationship that could be construed as a potential conflict of interest.

## Publisher’s Note

All claims expressed in this article are solely those of the authors and do not necessarily represent those of their affiliated organizations, or those of the publisher, the editors and the reviewers. Any product that may be evaluated in this article, or claim that may be made by its manufacturer, is not guaranteed or endorsed by the publisher.

## Footnotes

^1^ https://genome.ucsc.edu

^2^ http://great.stanford.edu/public/html/demo.php

^3^ http://www.webgestalt.org
