# Supplementary figures and images for: Significant Upregulation of HERV-K (HML-2) Transcription Levels in Human Lung Cancer and Cancer Cells
Source: Front Microbiol. 2022 Mar 10;13:850444. doi: 10.3389/fmicb.2022.850444 (PMC8960717; doi:10.3389/fmicb.2022.850444)

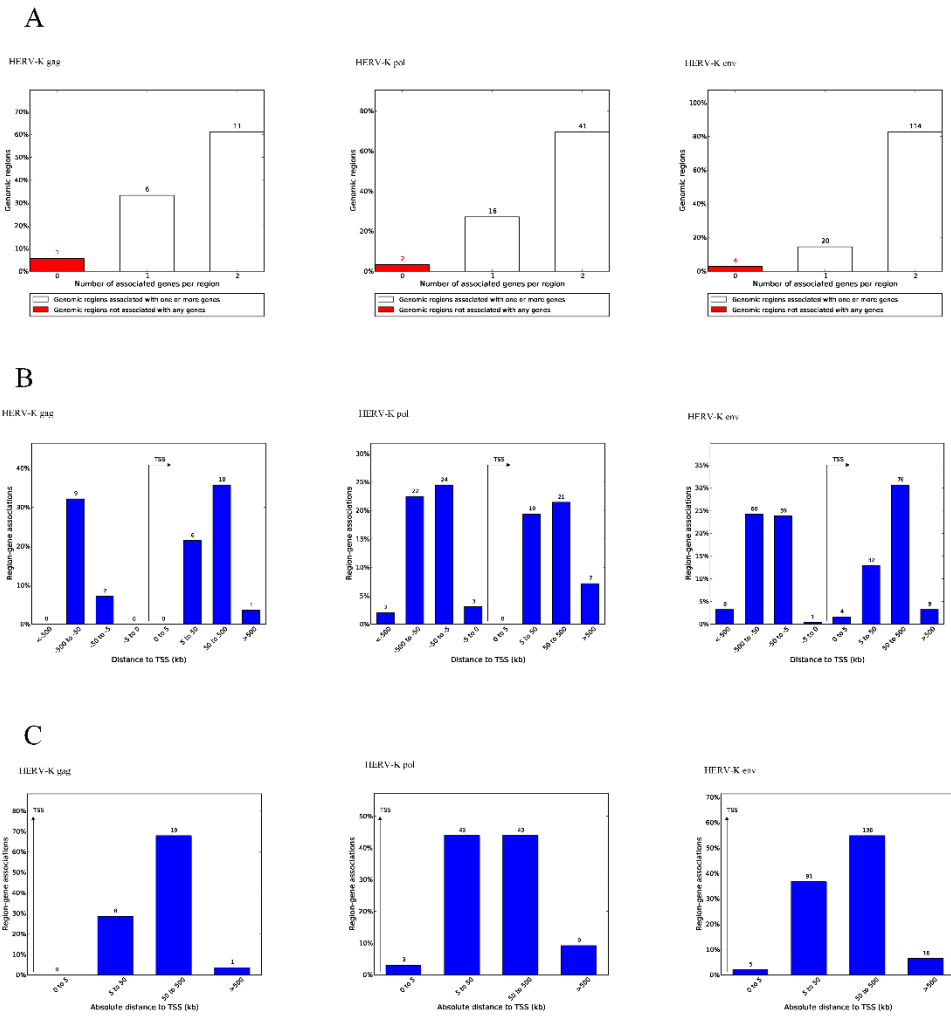

Supplement: Supplementary file 1 [file Image_1.pdf]
