# Supplementary material for: Significant Upregulation of HERV-K (HML-2) Transcription Levels in Human Lung Cancer and Cancer Cells
Source: Front Microbiol. 2022 Mar 10;13:850444. doi: 10.3389/fmicb.2022.850444 (PMC8960717; doi:10.3389/fmicb.2022.850444)
Supplement: Supplementary file 2 [file Table_1.pdf]

Supplementary Table 1. HERV-K (HML-2) gene annotation.

GREAT version 4.0.4

Species assembly: hg38

Association rule: Basal+extension: 5000 bp upstream, 1000 bp downstream, 1000000 bp max extension, curated regulatory domains included

GAG-gene to region

ABCC2	unnamed (+44327)
AGTR1	unnamed (-163470)
ALG1L	unnamed (+42503)
ANO2	unnamed (+110299)
ATP23	unnamed (+393471)
BOC	unnamed (-180952)
CEP126	unnamed (-218086)
DDX6	unnamed (+62640)
DEFB107A	unnamed (+310238)
DEFB107B	unnamed (+9632)
DNMBP	unnamed (+182860)
FOXK1	unnamed (-92279), unnamed (-92089), unnamed (-83776)
KMT2E	unnamed (-262256)
LHFPL3	unnamed (+423267)
LHX8	unnamed (+250518)
LRIG3	unnamed (+585474)
MEI4	unnamed (+33849), unnamed (+34039)
MSTO1	unnamed (+23753)
NEPRO	unnamed (-11737)
NTF3	unnamed (+403820)
OSBPL11	unnamed (-296880)
SLC44A5	unnamed (+232132)
TREH	unnamed (-48635)
TRPC6	unnamed (-113001)
YY1AP1	unnamed (+54678)

GAG-region to gene

unnamed	FOXK1 (-92089)
unnamed	MEI4 (+34039)
unnamed	OSBPL11 (-296880), ALG1L (+42503)
unnamed	ANO2 (+110299), NTF3 (+403820)
unnamed	CEP126 (-218086), TRPC6 (-113001)

unnamed	DEFB107B (+9632), DEFB107A (+310238)
unnamed	KMT2E (-262256), LHFPL3 (+423267)
unnamed	FOXK1 (-92279)
unnamed	FOXK1 (-83776)
unnamed	MEI4 (+33849)
unnamed	AGTR1 (-163470)
unnamed	BOC (-180952), NEPRO (-11737)
unnamed	NONE
unnamed	ATP23 (+393471), LRIG3 (+585474)
unnamed	ABCC2 (+44327), DNMBP (+182860)
unnamed	MSTO1 (+23753), YY1AP1 (+54678)
unnamed	TREH (-48635), DDX6 (+62640)
unnamed	SLC44A5 (+232132), LHX8 (+250518)

POL-gene to region

ABCC2	unnamed (+40356), unnamed (+40476)
ALG10	unnamed (+601338), unnamed (+601458)
ALG1L	unnamed (+38338)
ASRGL1	unnamed (+39526), unnamed (+39646)
ATP23	unnamed (+389508), unnamed (+389628)
ATP4A	unnamed (-11552)
BOC	unnamed (-184806)
C6orf226	unnamed (-6650)
C8orf33	unnamed (-27325)
CD48	unnamed (+15149)
CDH6	unnamed (-703480), unnamed (-703360)
CEP126	unnamed (-214117)
CEP97	unnamed (-26365)
CTAG1A	unnamed (+27045)
CTAG1B	unnamed (+7072)
DDX6	unnamed (+66483)
DEFB107A	unnamed (+314092), unnamed (+314212)
DEFB107B	unnamed (+5658), unnamed (+5778)
DGCR2	unnamed (+177940), unnamed (+178060)
DNMBP	unnamed (+186711), unnamed (+186831)
DUX4	unnamed (-63940)
EDA2R	unnamed (+151356)
ENSA	unnamed (-4111), unnamed (-3991)
ENSG00000283758	unnamed (-11050)
FOXK1	unnamed (-96254), unnamed (-96134), unnamed (-87750), unnamed (-87630)
FRG2	unnamed (-82580)

GBP1	unnamed (-22886)
GBP2	unnamed (+37868)
HCN1	unnamed (-307160)
HEPH	unnamed (+302125)
HOXB1	unnamed (+47424)
LCN15	unnamed (-18191)
LIPH	unnamed (-13392), unnamed (-13272)
LRIG3	unnamed (+589317), unnamed (+589437)
MEI4	unnamed (+29999)
MSTO1	unnamed (+19778), unnamed (+19898)
NEPRO	unnamed (-7883)
OSBPL11	unnamed (-301045)
POGK	unnamed (-231023)
PPP1R2P3	unnamed (-189495), unnamed (-189375)
PRAMEF13	unnamed (-14601)
PRAMEF14	unnamed (-11350)
PRAMEF18	unnamed (+13375)
PRAMEF19	unnamed (+13209)
PRAMEF27	unnamed (+40263)
PRAMEF6	unnamed (-68648)
PRKCQ	unnamed (-247625), unnamed (-247505)
PRODH	unnamed (-7961), unnamed (-7841)
PTCRA	unnamed (-18523)
RPL24	unnamed (-10778)
SCGB1A1	unnamed (-42061), unnamed (-41941)
SENP2	unnamed (-20289), unnamed (-20169)
SFMBT2	unnamed (+583560), unnamed (+583680)
SGCD	unnamed (+334286), unnamed (+334406)
SKAP1	unnamed (-53383)
SLAMF1	unnamed (-49724)
TMEM121	unnamed (+147699)
TMEM141	unnamed (-8651)
TMPRSS15	unnamed (-160427), unnamed (-160307)
TREH	unnamed (-44792)
TRIM27	unnamed (+234782)
TRPC6	unnamed (-116970)
UCK2	unnamed (+781535)
UGT2B15	unnamed (+70875)
UGT2B17	unnamed (-31226)
YY1AP1	unnamed (+58533), unnamed (+58653)
ZBED9	unnamed (-101872)
ZNF16	unnamed (-74165)
ZNF420	unnamed (+31760)

ZNF486	unnamed (+115119)
ZNF525	unnamed (-4660)
ZNF585A	unnamed (+62501)
ZNF737	unnamed (+283342)

POL-region to gene

unnamed	CTAG1B (+7072), CTAG1A (+27045)
unnamed	EDA2R (+151356), HEPH (+302125)
unnamed	LCN15 (-18191), TMEM141 (-8651)
unnamed	ZNF16 (-74165), C8orf33 (-27325)
unnamed	DEFB107B (+5778), DEFB107A (+314092)
unnamed	FOXK1 (-96134)
unnamed	FOXK1 (-87630)
unnamed	MEI4 (+29999)
unnamed	PTCRA (-18523), C6orf226 (-6650)
unnamed	PPP1R2P3 (-189375), SGCD (+334406)
unnamed	HCN1 (-307160)
unnamed	CDH6 (-703360)
unnamed	FRG2 (-82580), DUX4 (-63940)
unnamed	SENP2 (-20169), LIPH (-13392)
unnamed	BOC (-184806), NEPRO (-7883)
unnamed	TMPRSS15 (-160427)
unnamed	ATP4A (-11552), ENSG00000283758 (-11050)
unnamed	NONE
unnamed	ATP23 (+389628), LRIG3 (+589317)
unnamed	ALG10 (+601458)
unnamed	TREH (-44792), DDX6 (+66483)
unnamed	SCGB1A1 (-41941), ASRGL1 (+39646)
unnamed	ABCC2 (+40476), DNMBP (+186711)
unnamed	PRKCQ (-247625), SFMBT2 (+583560)
unnamed	POGK (-231023), UCK2 (+781535)
unnamed	MSTO1 (+19898), YY1AP1 (+58533)
unnamed	ENSA (-4111)
unnamed	PRAMEF6 (-68648), PRAMEF27 (+40263)
unnamed	ZBED9 (-101872), TRIM27 (+234782)
unnamed	UGT2B17 (-31226), UGT2B15 (+70875)
unnamed	CEP97 (-26365), RPL24 (-10778)
unnamed	PRODH (-7841), DGCR2 (+178060)
unnamed	ZNF525 (-4660)
unnamed	ZNF486 (+115119), ZNF737 (+283342)
unnamed	TMEM121 (+147699)
unnamed	PRAMEF13 (-14601), PRAMEF18 (+13375)

unnamed	PRAMEF14 (-11350), PRAMEF19 (+13209)
unnamed	SKAP1 (-53383), HOXB1 (+47424)
unnamed	NONE
unnamed	PRODH (-7961), DGCR2 (+177940)
unnamed	CEP126 (-214117), TRPC6 (-116970)
unnamed	SLAMF1 (-49724), CD48 (+15149)
unnamed	DEFB107B (+5658), DEFB107A (+314212)
unnamed	FOXK1 (-96254)
unnamed	FOXK1 (-87750)
unnamed	PPP1R2P3 (-189495), SGCD (+334286)
unnamed	CDH6 (-703480)
unnamed	SENP2 (-20289), LIPH (-13272)
unnamed	TMPRSS15 (-160307)
unnamed	ZNF420 (+31760), ZNF585A (+62501)
unnamed	ATP23 (+389508), LRIG3 (+589437)
unnamed	ALG10 (+601338)
unnamed	SCGB1A1 (-42061), ASRGL1 (+39526)
unnamed	ABCC2 (+40356), DNMBP (+186831)
unnamed	PRKCQ (-247505), SFMBT2 (+583680)
unnamed	MSTO1 (+19778), YY1AP1 (+58653)
unnamed	ENSA (-3991)
unnamed	GBP1 (-22886), GBP2 (+37868)
unnamed	OSBPL11 (-301045), ALG1L (+38338)

ENV-gene to region

ABCC2	unnamed (+39134)
ADCY10	unnamed (+142592)
ADCYAP1R1	unnamed (+101269)
ADGRB3	unnamed (+486155), unnamed (+796938)
AIPL1	unnamed (+259479)
AKAP11	unnamed (+204320)
AKAP9	unnamed (-51669), unnamed (+86924)
ALG1L	unnamed (+35750), unnamed (+35889)
ALKAL1	unnamed (-7145)
ALOX5	unnamed (-25537)
ANOS1	unnamed (+229793)
APCDD1	unnamed (+51322)
ARHGEF38	unnamed (-52064)
ASRGL1	unnamed (+32070)
ATG16L2	unnamed (+96019)
ATP23	unnamed (+386918), unnamed (+387062)
ATP4A	unnamed (-8992), unnamed (-8848)

BOC	unnamed (-187079)
C16orf72	unnamed (+74927), unnamed (+258447)
C2CD4C	unnamed (+22560)
C3orf38	unnamed (+154075)
C6orf226	unnamed (-3914)
CALML4	unnamed (-17064)
CASP6	unnamed (+25140)
CCDC180	unnamed (-177406)
CCPG1	unnamed (-37297)
CD48	unnamed (+12852), unnamed (+12996)
CDH6	unnamed (-705925)
CEP126	unnamed (-211668)
CEP97	unnamed (-24092), unnamed (-23948)
CES5A	unnamed (-33822)
CHMP3	unnamed (+18622)
CLN6	unnamed (+7118)
COL22A1	unnamed (-547093), unnamed (-546949)
CPSF2	unnamed (+65626)
CSF3R	unnamed (-6725)
CTSV	unnamed (-90610)
CXCR3	unnamed (-58526)
CYP51A1	unnamed (+106739)
DAD1	unnamed (+488399)
DCC	unnamed (-979142)
DDX6	unnamed (+68754), unnamed (+68898)
DEFB107A	unnamed (+316661), unnamed (+316805)
DEFB107B	unnamed (+3065), unnamed (+3209)
DGCR2	unnamed (+175639), unnamed (+175783)
DNAAF4	unnamed (+62678)
DNMBP	unnamed (+188053)
DNMT3A	unnamed (-191929)
DPPA5	unnamed (+20922)
DRD2	unnamed (-48905)
DTNB	unnamed (+139115)
EIF1AY	unnamed (-22971)
EMB	unnamed (-167254)
ENSG00000228144	unnamed (-5229)
ENSG00000259680	unnamed (-568000)
ENSG00000283758	unnamed (-13754), unnamed (-13610)
EPC1	unnamed (-158512)
EPHA3	unnamed (-803706)
EPX	unnamed (+16444)
FAM174A	unnamed (+250609)

FAM19A3	unnamed (+8481)
FCHSD2	unnamed (+231934)
FNIP2	unnamed (-9323)
FOXK1	unnamed (-98847), unnamed (-98703), unnamed (-90343), unnamed (-90199)
FRMD6	unnamed (-285246)
FXYD4	unnamed (-127759)
GABBR2	unnamed (+244580)
GABRG1	unnamed (+915282)
GAPT	unnamed (+71575)
GDNF	unnamed (+316623)
GHITM	unnamed (-618755)
GNAL	unnamed (-74461)
GNAO1	unnamed (-201930)
GNPDA2	unnamed (-482204)
GOLPH3	unnamed (+133008)
GPCPD1	unnamed (+157369)
GREB1L	unnamed (-226046)
GRIK3	unnamed (+544122)
GRIN2A	unnamed (+832659)
HK3	unnamed (-85259)
HSPBAP1	unnamed (+68662)
IPMK	unnamed (+23397)
IPO5	unnamed (-178680)
IRAK3	unnamed (-13665)
ISL2	unnamed (+563118)
ITGB1	unnamed (+452197)
KCNK9	unnamed (+241970), unnamed (+242114)
KDM3A	unnamed (+104054)
KDM5D	unnamed (-807831)
KHDC1	unnamed (-23158)
LCN15	unnamed (-16852)
LCP1	unnamed (-72666)
LGALS4	unnamed (+5739)
LGALS7B	unnamed (+18414)
LHX8	unnamed (+253843), unnamed (+253987)
LIMCH1	unnamed (-47362)
LIPH	unnamed (-11115)
LMBRD1	unnamed (+364806), unnamed (+675589)
LRCH1	unnamed (-269246)
LRIG3	unnamed (+591883), unnamed (+592027)
MAGEH1	unnamed (-80388)
MAPK4	unnamed (-142677)

MCUB	unnamed (+118128)
MED30	unnamed (-93153)
MEI4	unnamed (+27315), unnamed (+27459)
MEX3C	unnamed (-163710)
MIS18BP1	unnamed (-382536)
MKS1	unnamed (+10124)
MNS1	unnamed (-75786)
MPZL1	unnamed (+49560)
MSANTD3	unnamed (-36149)
MSTO1	unnamed (+17477), unnamed (+17621)
MTERF1	unnamed (-8478)
MYRFL	unnamed (-18209)
NAPG	unnamed (-20084)
NEPRO	unnamed (-5610)
NEUROD6	unnamed (+187164)
NHSL2	unnamed (-233782)
NMUR1	unnamed (-8222)
NPFFR1	unnamed (+45085)
NRF1	unnamed (+33193)
NSUN3	unnamed (-17971)
NTRK2	unnamed (-143501)
NUF2	unnamed (+176354)
NUP37	unnamed (+30899)
OPHN1	unnamed (-45202)
OR13A1	unnamed (-39822)
OR13F1	unnamed (-269514)
OR4E2	unnamed (+447035)
OSBPL11	unnamed (-303633), unnamed (-303494)
OTP	unnamed (-121446)
PAGE3	unnamed (-106871)
PALMD	unnamed (-136995)
PARP14	unnamed (+44544)
PARP8	unnamed (-58317)
PDZD2	unnamed (+401932)
PLGRKT	unnamed (+3061)
PLPPR4	unnamed (+244995)
PPA1	unnamed (-5164)
PPA2	unnamed (-26477)
PPID	unnamed (-36419)
PPP1R2P3	unnamed (-191796), unnamed (-191652)
PRDX4	unnamed (-143837)
PRODH	unnamed (-10262), unnamed (-10118)
PROKR2	unnamed (-139288)

PTCHD1	unnamed (+189593)
PXN	unnamed (-12242)
RAB3C	unnamed (-20030)
RAB3IP	unnamed (+67705)
RAB6C	unnamed (-16526)
RALBP1	unnamed (-44558)
RAP2A	unnamed (+340773)
RASGEF1A	unnamed (-14146)
RB1CC1	unnamed (+141780)
RLN1	unnamed (-94944)
ROCK1	unnamed (-25696)
RPL24	unnamed (-13195), unnamed (-13051)
RTP5	unnamed (+192244)
SCAPER	unnamed (+5493)
SCGB1A1	unnamed (-49517)
SENP2	unnamed (-22446)
SGCD	unnamed (+331985), unnamed (+332129)
SIRT4	unnamed (-24308)
SKA1	unnamed (+42406)
SLAMF1	unnamed (-52021), unnamed (-51877)
SLC16A1	unnamed (+227163)
SLC24A4	unnamed (-136042)
SLC28A3	unnamed (-185594)
SLC30A8	unnamed (+292232)
SLC35G4	unnamed (+4899)
SLC44A5	unnamed (+228663), unnamed (+228807)
SMC2	unnamed (+140489)
ST8SIA4	unnamed (+117352)
STX19	unnamed (-16335)
SYT6	unnamed (-90310)
TBC1D2	unnamed (-208896)
TBCA	unnamed (+15153)
TEX10	unnamed (-40426)
TEX44	unnamed (-54184)
THEG	unnamed (-9940)
TMEM141	unnamed (-9990)
TMEM181	unnamed (-187744)
TMPRSS15	unnamed (-158010)
TMPRSS5	unnamed (+182079)
TMX1	unnamed (+126533)
TNFSF11	unnamed (-97680)
TREH	unnamed (-42521), unnamed (-42377)
TRIM26	unnamed (-8635)

TRIM33	unnamed (+267933)
TRIM39	unnamed (-104843)
TRIM60	unnamed (-30070)
TRIM61	unnamed (-24215)
TRPC6	unnamed (-119419)
TTC26	unnamed (+92684)
TUBA3C	unnamed (+345573)
TULP4	unnamed (+36204)
TWSG1	unnamed (+95684)
UBE2H	unnamed (+289678)
UBN2	unnamed (-5023)
UCHL1	unnamed (+233962)
UIMC1	unnamed (+21817)
USP9Y	unnamed (-248120)
VCAN	unnamed (-33498)
VCX3B	unnamed (+37563)
VSTM2A	unnamed (-97704)
WASHC3	unnamed (-27101)
WDR70	unnamed (+139992)
WSCD1	unnamed (+106600)
XRCC4	unnamed (+360374)
YIPF6	unnamed (-19316)
YY1AP1	unnamed (+60810), unnamed (+60954)
ZNF280D	unnamed (+192666)
ZNF431	unnamed (+117367)
ZNF492	unnamed (-53602)
ZNF708	unnamed (+70003)
ZNF98	unnamed (-158376)

ENV-region to gene

unnamed	COL22A1 (-547093), KCNK9 (+241970)
unnamed	DEFB107B (+3209), DEFB107A (+316661)
unnamed	FOXK1 (-98703)
unnamed	FOXK1 (-90199)
unnamed	MEI4 (+27459)
unnamed	KHDC1 (-23158), DPPA5 (+20922)
unnamed	PPP1R2P3 (-191652), SGCD (+332129)
unnamed	CDH6 (-705925)
unnamed	SENP2 (-22446), LIPH (-11115)
unnamed	BOC (-187079), NEPRO (-5610)
unnamed	RAB6C (-16526)
unnamed	ENSG00000283758 (-13610), ATP4A (-8992)

unnamed	NONE
unnamed	ENSG00000259680 (-568000)
unnamed	ATP23 (+387062), LRIG3 (+591883)
unnamed	TREH (-42521), DDX6 (+68754)
unnamed	MSTO1 (+17621), YY1AP1 (+60810)
unnamed	OSBPL11 (-303494), ALG1L (+35889)
unnamed	CEP97 (-24092), RPL24 (-13051)
unnamed	PRODH (-10118), DGCR2 (+175783)
unnamed	NONE
unnamed	CEP126 (-211668), TRPC6 (-119419)
unnamed	SLAMF1 (-51877), CD48 (+12996)
unnamed	SLC44A5 (+228807), LHX8 (+253843)
unnamed	NHSL2 (-233782), CXCR3 (-58526)
unnamed	PRDX4 (-143837), PTCHD1 (+189593)
unnamed	VCX3B (+37563), ANOS1 (+229793)
unnamed	TEX10 (-40426), MSANTD3 (-36149)
unnamed	TBC1D2 (-208896), GABBR2 (+244580)
unnamed	RLN1 (-94944), PLGRKT (+3061)
unnamed	UBN2 (-5023), TTC26 (+92684)
unnamed	AKAP9 (-51669), MTERF1 (-8478)
unnamed	AKAP9 (+86924), CYP51A1 (+106739)
unnamed	VSTM2A (-97704)
unnamed	ADCYAP1R1 (+101269), NEUROD6 (+187164)
unnamed	HK3 (-85259), UIMC1 (+21817)
unnamed	ST8SIA4 (+117352), FAM174A (+250609)
unnamed	OTP (-121446), TBCA (+15153)
unnamed	EMB (-167254), PARP8 (-58317)
unnamed	GOLPH3 (+133008), PDZD2 (+401932)
unnamed	TRIM60 (-30070), TRIM61 (-24215)
unnamed	NONE
unnamed	GNPDA2 (-482204), GABRG1 (+915282)
unnamed	OSBPL11 (-303633), ALG1L (+35750)
unnamed	CEP97 (-23948), RPL24 (-13195)
unnamed	EPHA3 (-803706), C3orf38 (+154075)
unnamed	PRODH (-10262), DGCR2 (+175639)
unnamed	PROKR2 (-139288), GPCPD1 (+157369)
unnamed	LGALS4 (+5739), LGALS7B (+18414)
unnamed	ZNF98 (-158376), ZNF492 (-53602)
unnamed	THEG (-9940), C2CD4C (+22560)
unnamed	GNAL (-74461), SLC35G4 (+4899)
unnamed	GNAO1 (-201930), CES5A (-33822)
unnamed	C16orf72 (+74927)
unnamed	SCAPER (+5493), ISL2 (+563118)

unnamed	CALML4 (-17064), CLN6 (+7118)
unnamed	MNS1 (-75786), ZNF280D (+192666)
unnamed	FRMD6 (-285246), TMX1 (+126533)
unnamed	MIS18BP1 (-382536)
unnamed	IPO5 (-178680), RAP2A (+340773)
unnamed	LRCH1 (-269246), LCP1 (-72666)
unnamed	TNFSF11 (-97680), AKAP11 (+204320)
unnamed	IRAK3 (-13665), ENSG00000228144 (-5229)
unnamed	DRD2 (-48905), TMPRSS5 (+182079)
unnamed	ATG16L2 (+96019), FCHSD2 (+231934)
unnamed	GHITM (-618755)
unnamed	IPMK (+23397)
unnamed	OR13A1 (-39822), ALOX5 (-25537)
unnamed	FXYD4 (-127759), RASGEF1A (-14146)
unnamed	SYT6 (-90310), TRIM33 (+267933)
unnamed	PALMD (-136995), PLPPR4 (+244995)
unnamed	SLC44A5 (+228663), LHX8 (+253987)
unnamed	USP9Y (-248120)
unnamed	PAGE3 (-106871), MAGEH1 (-80388)
unnamed	LCN15 (-16852), TMEM141 (-9990)
unnamed	OR13F1 (-269514), SMC2 (+140489)
unnamed	CCDC180 (-177406), CTSV (-90610)
unnamed	SLC28A3 (-185594), NTRK2 (-143501)
unnamed	COL22A1 (-546949), KCNK9 (+242114)
unnamed	ALKAL1 (-7145), RB1CC1 (+141780)
unnamed	DEFB107B (+3065), DEFB107A (+316805)
unnamed	NRF1 (+33193), UBE2H (+289678)
unnamed	FOXK1 (-98847)
unnamed	FOXK1 (-90343)
unnamed	TMEM181 (-187744), TULP4 (+36204)
unnamed	MEI4 (+27315)
unnamed	ADGRB3 (+486155), LMBRD1 (+675589)
unnamed	LMBRD1 (+364806), ADGRB3 (+796938)
unnamed	TRIM39 (-104843), TRIM26 (-8635)
unnamed	PPP1R2P3 (-191796), SGCD (+331985)
unnamed	RAB3C (-20030), GAPT (+71575)
unnamed	WDR70 (+139992), GDNF (+316623)
unnamed	CASP6 (+25140), MCUB (+118128)
unnamed	ARHGEF38 (-52064), PPA2 (-26477)
unnamed	LIMCH1 (-47362), UCHL1 (+233962)
unnamed	PARP14 (+44544), HSPBAP1 (+68662)
unnamed	TMPRSS15 (-158010)
unnamed	RTP5 (+192244)

unnamed	TEX44 (-54184), NMUR1 (-8222)
unnamed	CHMP3 (+18622), KDM3A (+104054)
unnamed	ENSG00000283758 (-13754), ATP4A (-8848)
unnamed	NONE
unnamed	ZNF708 (+70003), ZNF431 (+117367)
unnamed	DCC (-979142), MEX3C (-163710)
unnamed	MAPK4 (-142677), SKA1 (+42406)
unnamed	GREB1L (-226046), ROCK1 (-25696)
unnamed	NAPG (-20084), APCDD1 (+51322)
unnamed	RALBP1 (-44558), TWSG1 (+95684)
unnamed	MKS1 (+10124), EPX (+16444)
unnamed	WSCD1 (+106600), AIPL1 (+259479)
unnamed	C16orf72 (+258447), GRIN2A (+832659)
unnamed	CCPG1 (-37297), DNAAF4 (+62678)
unnamed	SLC24A4 (-136042), CPSF2 (+65626)
unnamed	OR4E2 (+447035), DAD1 (+488399)
unnamed	TUBA3C (+345573)
unnamed	SIRT4 (-24308), PXN (-12242)
unnamed	WASHC3 (-27101), NUP37 (+30899)
unnamed	MYRFL (-18209), RAB3IP (+67705)
unnamed	ATP23 (+386918), LRIG3 (+592027)
unnamed	TREH (-42377), DDX6 (+68898)
unnamed	ABCC2 (+39134), DNMBP (+188053)
unnamed	PPA1 (-5164), NPFFR1 (+45085)
unnamed	EPC1 (-158512), ITGB1 (+452197)
unnamed	MPZL1 (+49560), ADCY10 (+142592)
unnamed	NUF2 (+176354)
unnamed	MSTO1 (+17477), YY1AP1 (+60954)
unnamed	KDM5D (-807831), EIF1AY (-22971)
unnamed	OPHN1 (-45202), YIPF6 (-19316)
unnamed	MED30 (-93153), SLC30A8 (+292232)
unnamed	VCAN (-33498), XRCC4 (+360374)
unnamed	NSUN3 (-17971), STX19 (-16335)
unnamed	SLAMF1 (-52021), CD48 (+12852)
unnamed	FAM19A3 (+8481), SLC16A1 (+227163)
unnamed	PPID (-36419), FNIP2 (-9323)
unnamed	C6orf226 (-3914)
unnamed	DNMT3A (-191929), DTNB (+139115)
unnamed	SCGB1A1 (-49517), ASRGL1 (+32070)
unnamed	CSF3R (-6725), GRIK3 (+544122)
