# Supplementary material for: Significant Upregulation of HERV-K (HML-2) Transcription Levels in Human Lung Cancer and Cancer Cells
Source: Front Microbiol. 2022 Mar 10;13:850444. doi: 10.3389/fmicb.2022.850444 (PMC8960717; doi:10.3389/fmicb.2022.850444)
Supplement: Supplementary file 3 [file Table_2.docx]

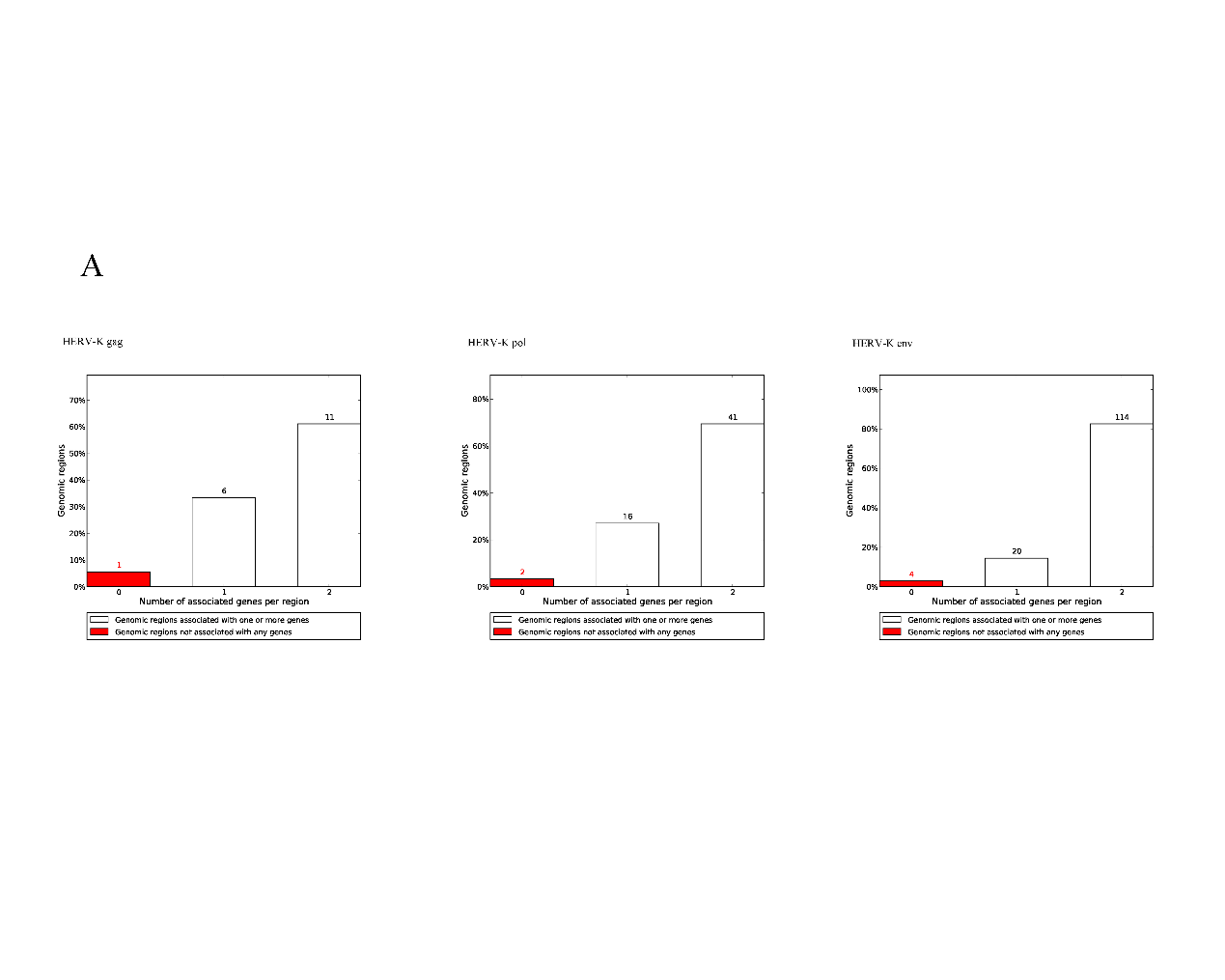


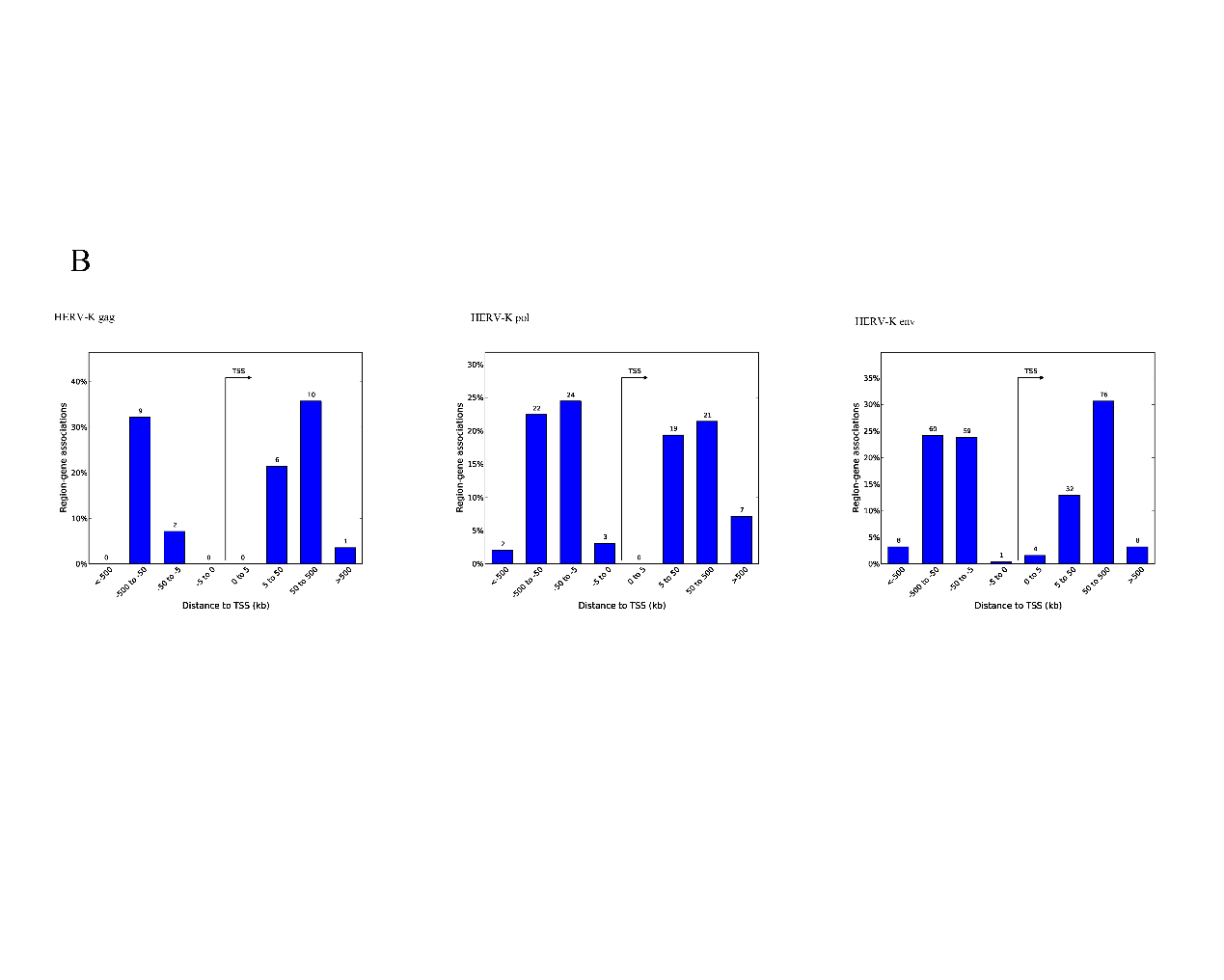


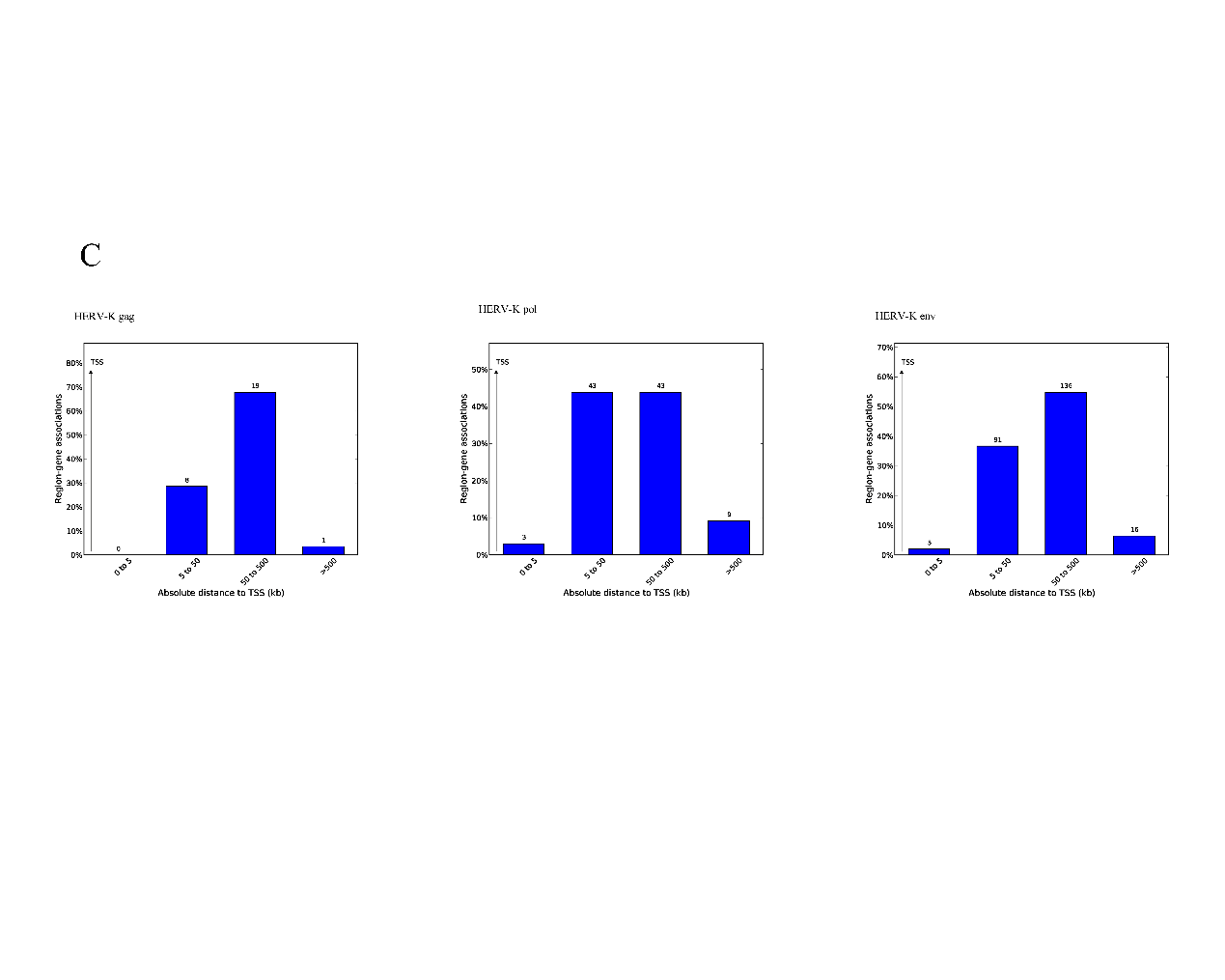


**S2 FIGURE. The associations between each HERV-K(HML-2) and the gene(s) it putatively regulates.** (A)The number of associated genes per HERV-K(HML-2). (B) Binned by orientation and distance to TSS. (C) Binned by absolute distance to TSS.
